# Dosimetric parameters and absolute monocyte count can predict the prognosis of acute hematologic toxicity in cervical cancer patients undergoing concurrent chemotherapy and volumetric-modulated arc therapy

**DOI:** 10.1186/s13014-022-02018-1

**Published:** 2022-03-05

**Authors:** Xiaoyong Xiang, Zhen Ding, Qi Zeng, Lingling Feng, Chunyan Qiu, Dongjie Chen, Jiawei Lu, Ning Li

**Affiliations:** 1 Department of Radiation Oncology, National Cancer Center/National Clinical Research Center for Cancer/Cancer Hospital & Shenzhen Hospital, Chinese Academy of Medical Sciences and Peking Union Medical College, Shenzhen, 518116 China; 2 Department of Radiation Oncology, National Cancer Center/National Clinical Research Center for Cancer/Cancer Hospital, Chinese Academy of Medical Sciences and Peking Union Medical College, Beijing, 100021 China

**Keywords:** Bone marrow, Acute hematological toxicity, Cervical cancer, Volumetric-modulated arc therapy, Absolute monocyte count

## Abstract

**Purpose:**

To explore clinical and dosimetric predictors of acute hematologic toxicity (HT) in cervical cancer patients treated with concurrent chemotherapy and volumetric-modulated arc therapy (VMAT).

**Methods and materials:**

We retrospectively reviewed the clinical data of 184 cervical cancer patients who had concurrent chemotherapy and VMAT. Hematological parameters were collected during the treatment period. The total pelvic bone (TPB) was delineated retrospectively for dose-volume calculations. To compare the differences between two groups, the normality test findings were used to run a paired-samples t-test or Wilcoxon signed-rank test. Pearson's correlation analysis or Spearman's correlation was used to testing the correlation between the two variables. Binary logistic regression analysis was used to analyze associations between HT and possible risk factors. The receiver operating characteristic curve(ROC) was used to evaluate the best cut-off point for dosimetric planning constraints.

**Results:**

The nadir of absolute monocyte count (AMC) was found to be positively correlated with the nadir of absolute white blood cells (WBC) count (r = 0.5378, 95% CI 0.4227–0.6357, *P* < 0.0001) and the nadir of absolute neutrophil count(ANC) (r = 0.5000, 95% CI 0.3794–0.6039, *P* < 0.0001). The AMC decreased and increased before the ANC and WBC. In multivariate logistic regression analysis, the chemotherapy regimens and the TPB_V20 were independent risk factors for developing grade ≥ 3 HT. The optimal TPB_V20 cut-off value identified by ROC curves and the Youden test was 71% (AUC = 0.788; 95% CI 0.722–0.845; *P* value < 0.001).

**Conclusions:**

The changing trend of AMC can be used as an effective predictor for the timing and severity of the ANC/WBC nadirs and prophylactic G-CSF administration. Maintain TPB_V20 < 71% and selecting single-agent cisplatin or carboplatin could significantly reduce grade ≥ 3 HT in cervical cancer patients undergoing concurrent chemoradiotherapy.

## Introduction

Cervical cancer is one of the most prevalent malignancies of the reproductive system in women, accounting for ~ 340,000 annual mortalities, according to the global cancer statistics released in 2020 [1]. Radical surgery is the first treatment strategy for early-stage cervical cancer. However, for patients with high-risk surgical-pathological factors, such as pelvic lymph node-positive, positive resection margins, and parametrial infiltration, or locally advanced cervical cancer, concurrent platinum-based chemoradiotherapy remains one of the main therapeutic options [2]. Previous studies have suggested that concurrent chemoradiotherapy (CCRT) improves treatment efficacy while increasing adverse reactions, with hematological toxicity (HT) being one of the main adverse effects during CCRT and follow-up [3, 4].

Patients with grade three or higher HT are more likely to develop life-threatening infections or febrile neutropenia, which may necessitate reducing CCRT doses or terminating it entirely, affecting therapeutic efficacy [5]. Therefore, in addition to reducing the severity and incidence of HT, improving pretreatment evaluation of leukopenia and neutropenic risk may help ensure that most patients continue to receive the optimal dose intensity chemoradiotherapy, boosting their chance to meet their treatment goals.

In cancer patients, approximately 34.5% of the active bone marrow is located in the pelvis bones, 16.6% in the lumbar vertebrae, and 4.5% in the proximal femur [6]. Because of the high radiosensitivity of hematopoietic stem cells, the pelvic bone is a potential organ at risk, especially in cervical cancer patients undergoing CCRT. Furthermore, we found that the absolute monocyte count (AMC) decreased and increased before the absolute neutrophil count (ANC)/absolute white blood cells (WBC) count, and that there was a clear correlation between the nadir of AMC and the severity of neutropenia/leukopenia.

Currently, cervical cancer is being treated with advanced treatment techniques, including intensity-modulated radiation therapy (IMRT), helical tomography radiotherapy (TOMO), or volumetric-modulated arc therapy (VMAT) regimens. A recent dosimetric study found that using VMAT with os coxae and lumbosacral spine as separate dose-volume constraints in patients with cervical cancer can minimize CCRT-associated HT [7]. However, there is no unified standard for limiting the optimal dosimetric parameters of pelvic bones marrow to reduce the incidence of severe HT [8, 9]. Therefore, the aim of this study was to explore the clinical and dosimetric predictors of HT in cervical cancer patients treated with CCRT in our institution. We also examined the predictive value of AMC for acute leukopenia/neutropenia.

## Materials and methods

### Patients

We retrospectively reviewed the clinical data of 184 cervical cancer patients who had CCRT at our institution between October 2018 and March 2021. All eligible patients had either newly diagnosed or recurrent cervical cancer that was confirmed using biopsy, and they had either radical or postoperative pelvic radiotherapy with concurrent weekly platinum-based chemotherapy. The pelvic radiotherapy was administered using an image-guided VMAT technique. Patients who had previously received pelvic radiotherapy or CCRT with extended field pelvic para-aortic irradiation, as well as those with incomplete data, were excluded from this study. Except for anemia, patients with grade ≥ 2 HT in the last two weeks before the CCRT, and patients with established bone marrow metastases, were also excluded. During the chemoradiotherapy period, the patients were assessed for HT at least once a week using complete blood counts. All patients in this study signed informed consent forms.

### Concurrent chemotherapy

All chemotherapy regimens were platinum-based. The regimens included TP (135–175 mg/m^2^ paclitaxel, D1; 50–70 mg/m^2^ cisplatin, D2-4; 21 day repeat, 1–2 cycles), weekly cisplatin (40 mg/m^2^, D1, 5–6 cycles), and weekly carboplatin (AUC 2, D1, 5–6 cycles). Chemotherapy regimens differed because the patient, their family, and physicians decidec on the specific regimen, with cisplatin as the preferred agent. Before each cycle of chemotherapy, all patients underwent routine hematological examination, including hematology, blood chemistry, renal function, and liver function. The next cycle of chemotherapy was administered if patients tolerated the previous cycle.

### Volumetric-modulated arc therapy

Typically, the patient was simulated in the prone position. If necessary, the patient was immobilized was performed in the supine position. A contrast-enhanced CT-based simulation was performed with a slice thickness of 5 mm when patients were moderately filling the bladder and emptying the rectum. The CT scan covered the area from the lower edge of the T10 vertebrae to the 5 cm below the ischial tuberosity. The clinical target area (CTV) included the entire cervix, uterus, partial vaginal, vesicovaginal and rectovaginal spaces, gross tumor, parametria, and regional lymph nodes. Lymph node metastasis was diagnosed using imaging test results (such as MRI, CT, and PET-CT) or pathological biopsy. The planning target volume (PTV) included the CTV and a uniform three-dimensional expansion of 5 mm.

All the patients in this study underwent pelvic volumetric-modulated arc therapy. The pelvic radiation dose was 45–50.4 Gy in 1.8 Gy daily fractions, and the dose delivered to the affected lymph nodes was 58.8–60.2 Gy in 2.1–2.15 Gy daily fractions. The following organs at risk (OARs) were delineated for dose-volume calculations: bladder, spinal cord, femoral heads, rectum, small intestine, sigmoid colon, and bowel bag. The pelvic bone marrow was not included in the OARs but was delineated retrospectively for dose-volume calculations. Cone-beam computed tomography was used to minimize the daily setup error. Vaginal brachytherapy was dependent on the specific circumstances of the patient, and the dose to pelvic bone marrow from brachytherapy was considered insignificant. In the final week of external beam radiotherapy, patients who accepted radical radiotherapy usually started receiving four to five fractions of 3D image-guided high dose rate brachytherapy (intracavitary or interstitial or a combination of both; one to two fractions per week). We aimed for cumulative EBRT and brachytherapy doses of > 85 Gy (EQD2) for HR-CTV D90.

### Bone marrow delineation

**Fig. 1:**
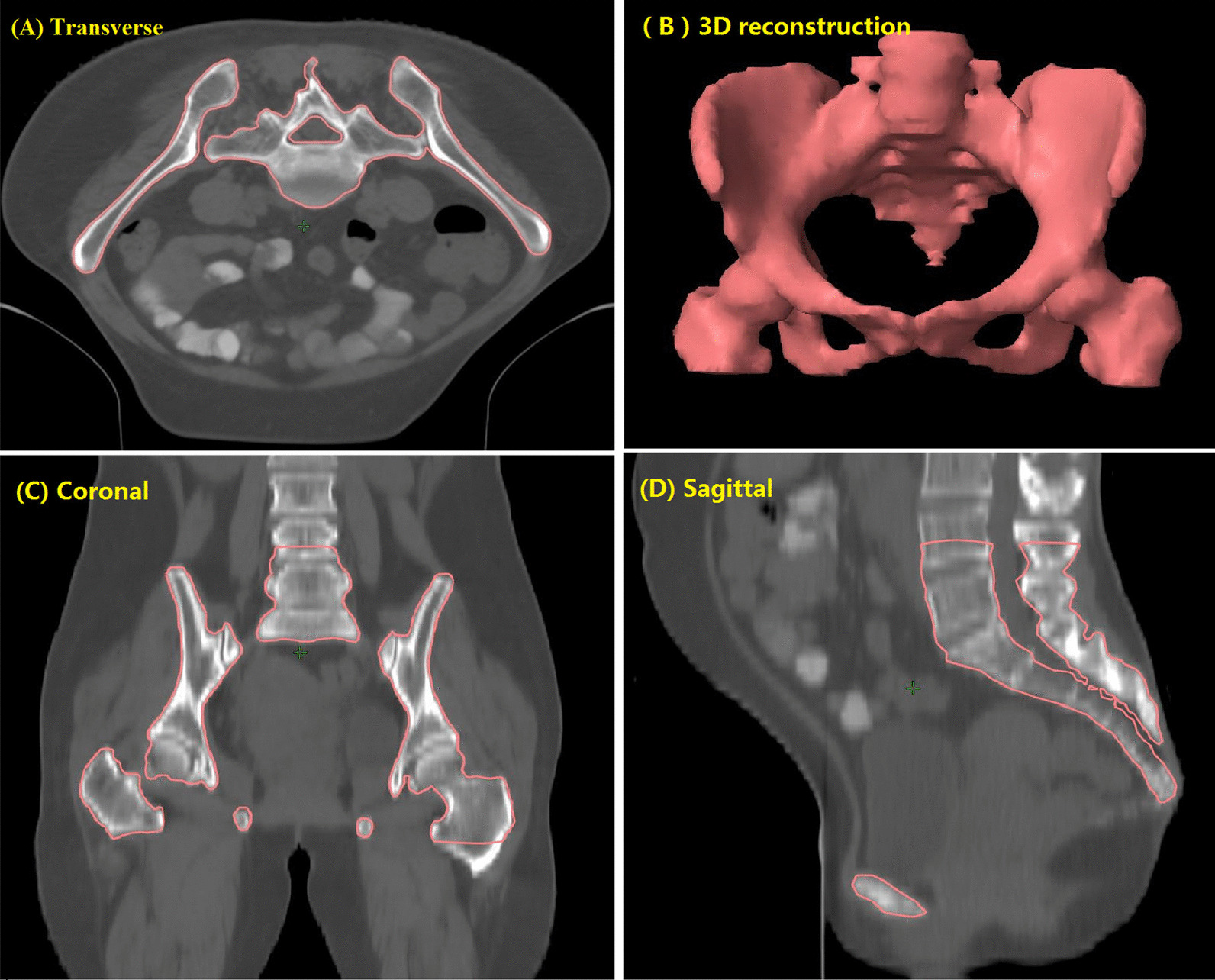
Typical images showing contours for the total pelvic bone (TPB) [9]

All bone marrow contours were retrospectively delineated manually according to Mell et al. [9]. To ensure repeatability and limit inter-observer variations, all the external contours of the bones within the PTV coverage (defined as from the centrum 2 cm above the upper boundary of PTV to the ischial tuberosities) were retrospectively delineated as a proxy for the bone marrow rather than the low-density areas within the bones by a single radiation oncologist and subsequently reviewed by another senior radiation oncologist. The contour of the total pelvic bone (TPB) is shown in Fig. 1.

Dose-volume histograms (DVHs) corresponding to the delivered VMAT plan were used to assess the dose volumes (V10, V20, V30, V40, and V50, respectively) received by the TPB.

### Hematologic toxicity

All patients agreed to a routine blood examination before receiving external irradiation or platinum-based chemotherapy. Blood routine tests should be taken at least once a week during the period of CCRT and within two weeks after completing external radiation therapy. However, the frequency of routine blood examination can be increased if necessary. The day of CCRT initiation was defined as day 0 (D0), while the first day after CCRT initiation was defined as day 1 (D1). The days were calculated from D0, when AMC, ANC, PLT, WBC, and HGB were at their minimal level. Using a similar approach, the days from the D1 were calculated when AMC, ANC, and WBC increased from the nadir to ≥ normal value for the first time.

The HT was graded according to the Common Terminology Criteria for Adverse Events (CTCAE) v5.0. Whether the patients were treated with granulocyte-monocyte colony-stimulating factors, red cell transfusion, or platelet transfusion was based on the clinical judgment of the attending physicians. Generally, the criteria were as follows: absolute neutrophil counts < 1000/mm^3^ or absolute WBC count < 2000/mm^3^, haemoglobin < 60 g/L, and PLT < 20 × 109/L.

### Statistical analysis

Data analysis was performed using SPSS (IBM SPSS 23.0, SPSS Inc). Descriptive statistics were generated for relevant clinical and dosimetric parameters. The Shapiro–Wilk test was used to determine the normality of data distribution. Data with to normal distribution were expressed as mean ± standard deviation, whereas the rest were expressed as the median and interquartile range (IQR). Categorical data were assessed and described as frequencies and percentages. To compare the differences between two groups, the normality test findings were used to run a paired-samples t-test or Wilcoxon signed-rank test. Pearson's correlation analysis or Spearman's correlation was used to testing the correlation between the two variables, with *P* < 0.05 considered significant. Possible risk factors for grade ≥ 3 HT were analyzed using binary logistic regression analysis, and variable with *P* values < 0.05 in the binary logistic regression analysis were entered into the multiple logistic regression analysis. The receiver operating characteristic (ROC) curve was used to evaluate the area under the ROC curve (AUC), best cut-off point, specificities, and sensitivities. Statistical significance was considered when *P* < 0.05.

## Results

### Patient characteristics

In this study, 184 cervical cancer patients received pelvic radiotherapy using an image-guided VMAT technique. Furthermore, the patients were treated with platinum-based chemotherapy, with 42 (22.8%) receiving TP, 111 (60.3%) receiving weekly cisplatin, and 31 (16.9%) receiving weekly carboplatin. Every patient received at least one cycle of concurrent chemotherapy (with a median of two cycles, range from one to six cycles). The basic clinical characteristics of the patients are shown in Table 1.

**Table 1:** Basic clinical characteristics of the patients

Patients (n)		184
Age (years)	Median, Mean (Range, SD)	54, 53.3 (31–81, 10.3)
BMI (kg/m^2^)	Median, Mean (Range, SD)	22.59, 23.1 (16.2–33.8, 3.3)
Duration of EBRT (days)	Median, Mean (Range, SD)	37, 38 (29–52, 3.9)
RT dose to pelvis (Gy)	Median, Mean (Range, SD)	45, 47.4 (45–50.4, 2.6)
Cycles of chemotherapy	Median, Mean (Range, SD)	2, 2.9 (1–6, 1.5)
Clinical stage FIGO2018 (n, %)	IB–IIB IIIA–IVB	98 (53.3) 86 (46.7)
Histology, n (%)	Squamous carcinoma Adenocarcinoma	166 (90.2) 18 (9.8)
Differentiation degree, n (%)	High–moderate Lower / Unknown	85 (46.2) 99 (53.8)
PTV dose-pelvis (n, %)	45 Gy 48.6 Gy–50.4 Gy	99 (53.8) 85 (46.2)
Chemotherapy regimen (n, %)	TP Carboplatin Cisplatin	42 (22.8) 31 (16.9) 111 (60.3)

BMI, body mass index; EBRT, pelvic external-beam radiotherapy; SD, standard deviation; RT, radiation therapy; TP, paclitaxel + cisplatin

### Dosimetric parameters of the pelvic bone

The dosimetric parameters were subjected to a descriptive statistical analysis, and their mean values, median values, maximum, minimum, 25th percentile, 75th percentile, and standard deviations were recorded. Table 2 shows the dosimetric parameters for the total pelvic bone marrow.

**Table 2:** Descriptive statistics of dosimetric parameters of the total pelvic bones

Parameter		Mean	Median	Min–max	Q1–Q3	SD
TPB	Volume (cm^3^) V10 (10%) V20 (20%) V30 (30%) V40 (40%) V50 (50%)	1189 90.39 71.53 45.41 23.32 5.14	1165 90.00 70.74 45.00 23.00 3.00	886–2219 49.00–100.00 27.23–92.90 14.00–68.00 6.00–46.00 0.00–26.00	1070–1266 88.00–94.00 66.95–76.89 39.00–51.00 18.00–27.00 0.00–10.00	177.6 5.780 7.977 8.832 6.617 5.422

V10, V20, V30, V40, V50 = volume receiving 10,20, 30, 40, 50 Gy; TPB, the total pelvic bones; Max, maximum; Min, minimum; Q1, 25th percentile; Q3, 75th percentile; SD, standard deviation

### Hematological baselines and nadirs

**Fig. 2:**
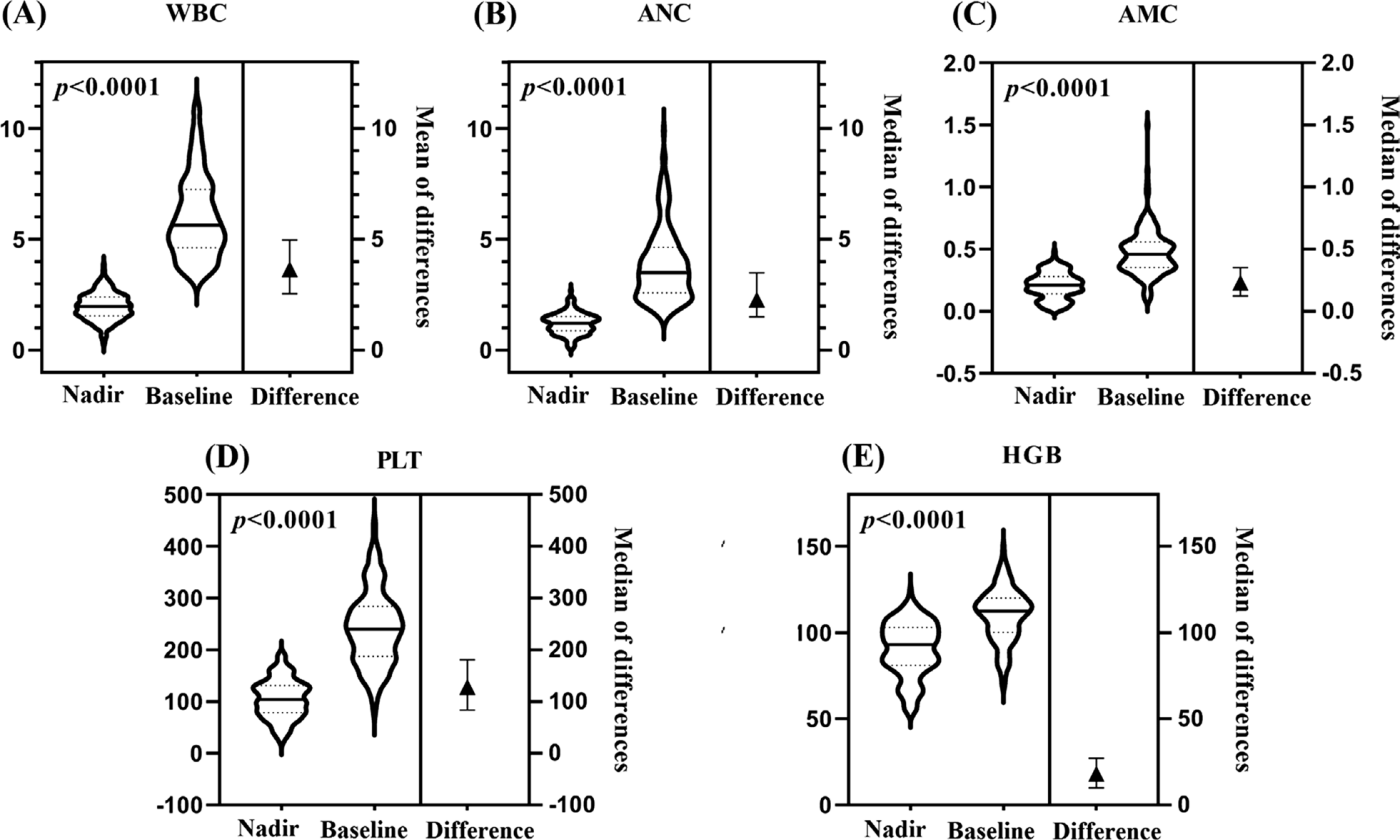
Descriptive statistics of hematological baselines and nadirs

Compared to the hematological baselines, all blood cell counts declined to varying degrees throughout the study period (*P* < 0.001). The median of WBC, ANC, HGB, AMC, and PLT counts at the nadirs were 1.99 × 10^9^/L (Q1–Q3, 1.62–2.37), 1.21 × 10^9^/L (Q1–Q3, 0.91–1.51), 90.00 g/L (Q1–Q3, 80–103), 0.21 × 10^9^/L (Q1–Q3, 0.14–0.28), and 104.00 10^9^/L (Q1–Q3, 78.25–131.00), respectively. The WBC, ANC, HGB, AMC, and PLT counts decreased by 64.72%, 64.86%, 16.00%, 50.00%, and 53.44%, respectively (Fig. 2).

### Hematologic toxicity

At baseline, 26 patients (14.13%) had grade 1 leukopenia, 11 patients (5.98%) had grade 1 neutropenia, and two patients (1.09%) had grade 1 thrombocytopenia. Moreover, anemia was more prevalent at baseline than other HT. At baseline, 50 patients (27.17%) had grade 1, 20 patients (10.89%) had grade 2, and seven patients (3.08%) had grade 3 anemia. The percentages of grade 3 or grade 4 leukopenia, neutropenia, thrombocytopenia, and anemia during CCRT were 52.17%, 30.43%, 6.52%, and 22.83%, respectively. Fifty-six (30.4%) patients received colony-stimulating factors, four (2.2%) received platelet transfusion, and seventeen (9.2%) received packed RBC transfusion. Overall, 111 patients (60.33%) did experience any grade 3+ HT. Further information is given in Table 3.

**Table 3:** Hematological toxicity graded according to hematological nadirs and baselines

Hematologic toxicity		Grade 0 (n, %)	Grade 1 (n, %)	Grade 2 (n, %)	Grade 3 (n, %)	Grade 4 (n, %)
Baselines	Leukopenia	158 (85.87)	26 (14.13)	0 (0)	0 (0)	0 (0)
	Neutropenia	173 (94.02)	11 (5.98)	0 (0)	0 (0)	0 (0)
	Thrombocytopenia	182 (98.91)	2 (1.09)	0 (0)	0 (0)	0 (0)
	Anemia	107 (58.15)	50 (27.17)	20 (10.89)	7 (3.80)	0 (0)
Nadirs	Leukopenia	0 (0)	9 (4.89)	79 (42.93)	88 (47.83)	8 (4.35)
	Neutropenia	9 (4.89)	41 (22.28)	78 (42.39)	46 (25.00)	10 (5.43)
	Thrombocytopenia	100 (53.8)	45 (24.46)	27 (14.67)	11 (5.98)	1 (0.54)
	Anemia	17 (9.24)	62 (33.70)	63 (34.24)	29 (15.76)	13 (7.07)

### The changing trend of blood cell counts

**Fig. 3:**
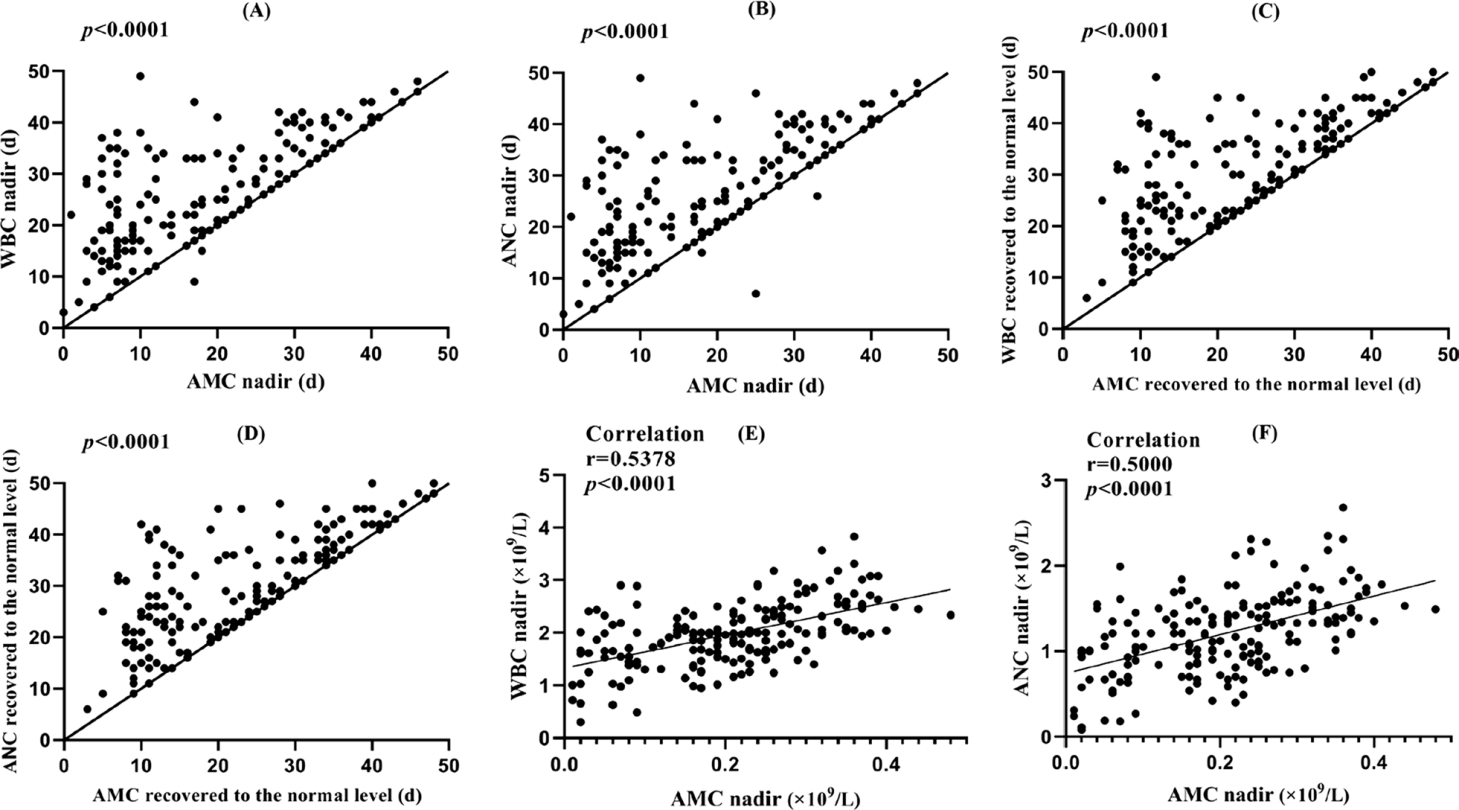
The changing tendency and correlation between AMC and ANC/WBC

The median time to the nadir was 26 days (Q1–Q3, 19–34 days) for the WBC and ANC, 20 days (Q1–Q3, 8 to –29 days) for the AMC, 29 days (Q1–Q3, 20–39 days) for the PLT, and 35 days (Q1–Q3, 24–42 days) for the HGB. When compared to the WBC and ANC, the AMC was the first to decreased to the nadir (median = five days, Q1–Q3 = 0 to 11 days, *P* < 0.001, Fig. 3A and B). The time it took for AMC to increase from the nadir to ≥ normal value for the first time was less than for WBC (median = four days, Q1–Q3 = 0 to 11 days, *P* < 0.001) and ANC (median = three days, Q1–Q3 = 0 to 10 days, *P* < 0.001, Fig. 3C and D). We also assessed the relationship between variables using the Spearman rank correlation test. The nadir of AMC was found to be positively correlated with the nadir of WBC (*r* = 0.5378, 95% CI 0.4227–0.6357, *P* < 0.0001) and the nadir of ANC (*r* = 0.5000, 95% CI 0.3794–0.6039, *P* < 0.0001) (Fig. 3E and F).

All of the preceding analyses indicate that AMC decreased and increased before the ANC and WBC, and that the changing tendency of AMC had certain predictive value to the changing trend of WBC and ANC.

### Predictors of hematologic toxicity

The univariate and multiple binary logistic regression analyses were performed to identify any grade ≥ 3 HT predictors. In univariable binary logistic regression analysis, the chemotherapy regimen and the total pelvic bone (TPB_V10, TPB_V20, TPB_V30, TPB_V40, and TPB_V50) were associated with any grade ≥ 3 HT (Table 4).

**Table 4:** Univariate logistic regression analysis of factors associated with the development of any grade ≥ 3 hematologic toxicity

Parameter	Odds ratio	95% CI	*P* value
Age (years)	1.000	0.972–1.029	0.991
BMI (kg/m^2^)	0.982	0.898–1.074	0.982
Duration of EBRT (days)	1.069	0.987–1.158	0.102
Clinical stage (FIGO2018)	1.759	0.964–3.208	0.066
Histology	0.559	0.232–1.345	0.194
Differentiation degree	0.789	0.431–1.445	0.443
PTV dose-pelvis	1.696	0.930–3.094	0.085
Cycles of chemotherapy	1.056	0.861–1.295	0.600
Volume (cm^3^)	1.002	1.000–1.004	0.053
Chemotherapy regimen			
TP			0.027*
Carboplatin	0.257	0.094–0.701	0.008*
Cisplatin	0.442	0.198–0.987	0.046*
TPB_V10	1.197	1.109–1.292	< 0.001*
TPB_V20	1.176	1.109–1.248	< 0.001*
TPB_V30	1.102	1.057–1.149	< 0.001*
TPB_V40	1.114	1.055–1.177	< 0.001*
TPB_V50	1.142	1.069–1.221	< 0.001*

BMI, body mass index; EBRT, pelvic external-beam radiotherapy; TPB_V10, TPB_V20, TPB_V30, TPB_V40, TPB_V50 = the total pelvic bones of volume receiving 10,20, 30, 40, 50 Gy; TP, paclitaxel + cisplatin. Bold indicates the significant values (*P < 0.05)

**Fig. 4:**
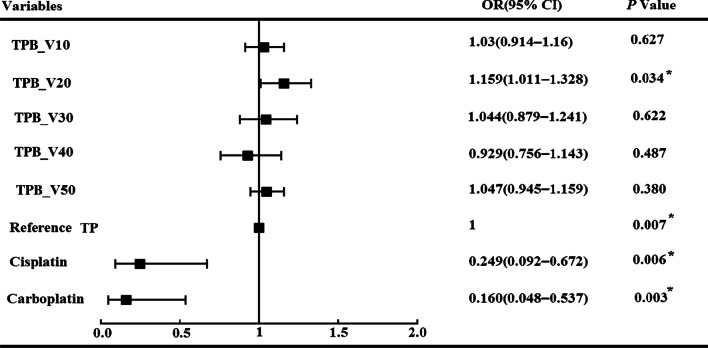
The results of multivariate binary logistic regression analysis for any grade ≥ 3 HT in patients

Subsequently, multivariate analysis was performed using all the significant factors in the univariate analysis. The results demonstrated that the chemotherapy regimens and TPB_V20 were independent factors. Patients who received the TP chemotherapy regimen were more likely to develop grade ≥ 3 HT than those who received cisplatin or carboplatin (76.2% vs. 58.6% vs. 45.2%). Furthermore, patients with increased TPB_V20 were more likely to develop grade ≥ 3 HT (OR 1.154; SE 0.69; *P* = 0.034; 95% CI 1.008–1.321). Hosmer–Lemeshow test indicated that the models fitted well (χ^2^ = 8.412, *P* value = 3.94 > 0.05). The detailed results are shown in Fig. 4.

**Fig. 5:**
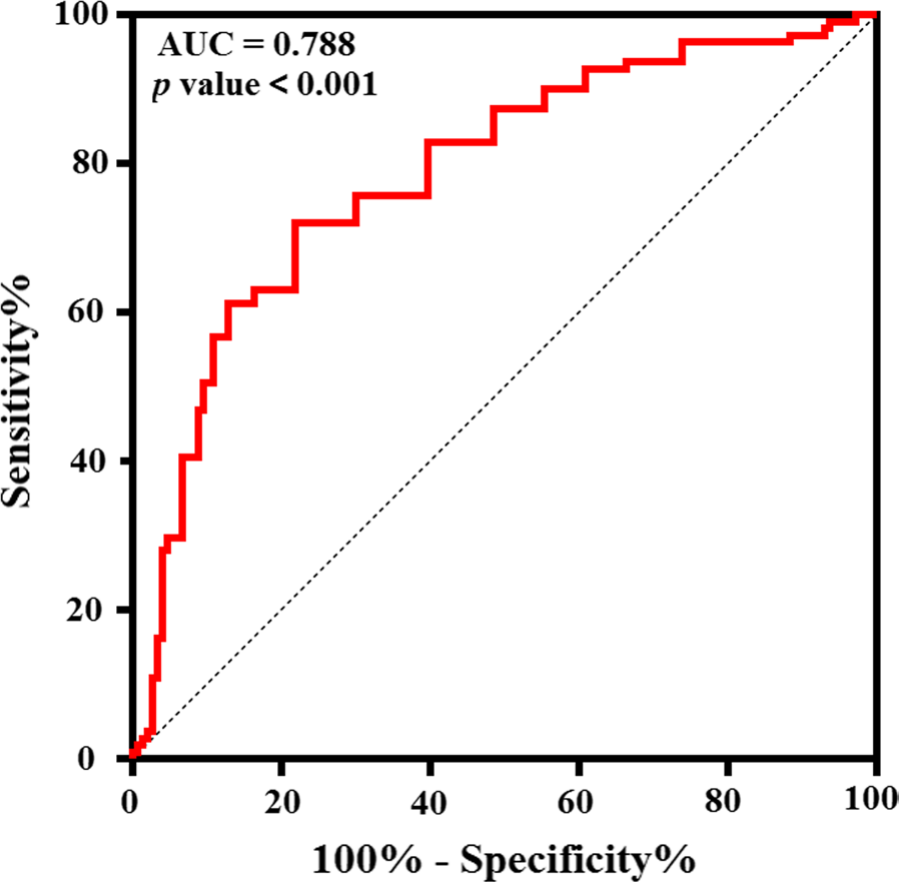
Receiver operating characteristic (ROC) curves for any grade ≥ 3 hematologic toxicity as a function of TPB_V20

The ROC curve for grade ≥ 3 HT versus TPB_V20 was analyzed to determine the optimal thresholds for dosimetric planning. The optimal TPB_V20 cut-off value determined by ROC curves and the Youden test was 71% (AUC = 0.788; 95% CI, 0.722–0.845; *P* value < 0.001, Fig. 5). Patients who received TPB_V20 ≥ 71% were more likely to develop grade ≥ 3 HT (84.1% vs. 38.5%, *P* < 0.001). The specificity and sensitivity for this threshold were 78.1% and 71.2%, respectively. The positive and negative predictive values for TPB_V20 ≥ 71% were 83.1% (95% CI 77.3%–87.7%) and 64.1% (95% CI 56.7%–70.8%), respectively.

## Discussion

Acute hematological toxicity (HT) is prevalent in cervical cancer patients who receive CCRT, especially grade ≥ 3 HT, which may result in lower CCRT doses or termination of CCRT altogether, affecting therapeutic efficacy [5]. To our knowledge, this is the first report to explore the predictive value of AMC in cervical cancer patients undergoing CCRT. We observed the AMC trend corresponded to the WBC/ANC during CCRT. The decrease and increase of AMC occurred before the ANC/WBC, and there was a significant correlation between the nadir of AMC and the severity of neutropenia/leukopenia. Furthermore, the findings in this study revealed quantifiable evidence of an association between TPB_V20 and acute grade ≥ 3 HT.

Multiple studies have established that prolonging the treatment time has a negative impact on disease-specific survival in cervical cancer patients who have undergone CCRT [10–12]. However, treatment time was mainly prolonged due to the acute HT, especially in patients with grade ≥ 3 neutropenia/leukopenia. These patients are more likely to develop life-threatening infections or febrile neutropenia, which could lead to a reduction in CCRT doses or termination of CCRT altogether, affecting the therapeutic efficacy. According to the ASCO recommendation, granulocyte colony-stimulating factor (G-CSF) can be used to prevent febrile neutropenia in high-risk patients [9]. However, treatment time and cost will be high in patients with grade ≥ 3 neutropenia/leukopenia despite the use of G-CSF. In the current clinical practice, risk assessment, control, and prevention of life-threatening infection or febrile neutropenia, are not standardized. Therefore, minimizing HT is critical when adopting more intense chemotherapy regimens for cervical cancer, and HT research should be strengthened.

Previous research has found statistically strong correlations between AMC and ANC during chemotherapy [13–18]. Kondo et al. showed that AMC < 150/microl after six to eight days of advanced lung cancer chemotherapy could be a predictor of grade 3 or higher neutropenia at three- or four-week intervals [13]. Subsequently, Oshita F et al. compared a group of 60 patients with unresectable lung cancer who were randomly assigned to prophylactic G-CSF administration when monocytopenia appeared (group A) or G-CSF administration when neutropenia (< 1,000/microl) or leukopenia (< 2000/microl) appeared after chemotherapy (group B) [14]. Their results indicated that prophylactic G-CSF administration can considerably shorten the duration (1.4 ± 1.7 days vs. 2.9 ± 1.9 days, *P* = 0.004) and frequency (48% vs. 83%, *P* = 0.002) of grade 3 neutropenia [14]. Furthermore, in another study of 75 patients with advanced lung cancer undergoing chemotherapy, the number of days that elapsed between the first day of chemotherapy and the median AMC nadir was shorter than that for AMC nadir (6 days vs. 12 days, *P* < 0.001); AMC at the nadir of 100/μL predicted an ANC at the nadir of < 1000/μL with a sensitivity of 83% and a specificity of 56% [15]. Similar results were reported in another study, which found that the changing trend of AMC was the same as that of ANC. The initial decrease / reaching the nadir / final increase days of AMC were significantly less than those of ANC [16]. Two more studies found a correlation between ANC and AMC in patients receiving chemotherapy [17, 18].

In this retrospective study, we evaluated the relationship between AMC and ANC/WBC, as well as the prognostic value of AMC for acute leukopenia/neutropenia in cervical cancer patients undergoing CCRT. The results revealed that AMC decreased and increased before ANC and WBC, and that the changing tendency of AMC had certain predictive value to the changing trend of WBC and ANC. The nadir of AMC was also found to be positively correlated with the nadir of WBC/ANC. During hematopoiesis, both neutrophils and monocytes are differentiated from the granulocyte–macrophage colony-forming cells, providing a good explanation for a positive correlation between AMC and ANC nadirs during CCRT. Therefore, based on the findings of previous research and this study, the increase or decrease in AMC can be used as an effective predictor for the timing and severity of the ANC/WBC nadirs and prophylactic G-CSF administration.

Additionally, all the blood cell counts of patients were reduced to varying degrees during the CCRT. The WBC, ANC, HGB, AMC, and PLT counts decreased by 64.72%, 64.86%, 16.00%, 50.00%, and 53.44%, respectively. The percentage of patients with grade 3 or grade 4 neutropenia, leukopenia, thrombocytopenia, and anemia during CCRT was 52.17%, 30.43%, 6.52%, and 22.83%, respectively. Overall, 111 patients (60.33%) experienced grade 3+ HT. We found that white blood cell and neutrophil counts declined significantly in most patients during CCRT. The current treatment option for stage IB-IVA cervical cancer is weekly cisplatin with radiotherapy. However, different results were obtained in various studies. For example, according to a meta-analysis by Petrelli F et al., platinum-based doublet chemotherapy combined with concurrent radiotherapy can improve the overall survival and disease-free survival compared to weekly cisplatin with radiotherapy. Nonetheless, severe HT was largely underreported in the reviewed publications [19]. In our study, weekly cisplatin and weekly carboplatin were considerably more effective than the TP regimen in reducing ≥ 3 HT (58.6% vs. 45.2% vs. 76.2%). Therefore, it is critical to identify the optimum chemotherapy regimens for individual patients in order to reduce the chemotherapy-related HT.

Several studies had analyzed the relationship between pelvic dosimetry parameters and HT in patients undergoing pelvic radiotherapy with respect to radiation doses. For example, Mell et al. analyzed 37 cervical cancer patients who received CCRT and found that the risk of developing grade 2 or higher leukopenia and neutropenia increased by a factor (odds ratio) of 2.09 if the TPB_V10 exceeds 90% [9]. According to Rose et al., HT increases as the total pelvic bone volume irradiated increases, and efforts to keep TPB_V10 < 95% and TPB_V20 < 76% may help avoid grade 3+ leukopenia [20]. Additionally, a recent study demonstrated that lower pelvis (V5 > 95% or V20 > 45%), TPB_V20 ≥ 65%, and the mean dose of iliac crests > 31 Gy were most significantly correlated with grade 4 HT [8]. Wan et al. demonstrated that lumbosacral spine V40 was most significantly correlated with grade ≥ 2 HT in rectal cancer patients, and they recommended dose constraints to the lumbosacral spine to be V40 < 60% [21]. Franco et al. found that a higher TPB_V20 was associated with lower WBC nadir in anal cancer patients, and that patients with lumbosacral spine V40 < 41% may reduce grade 3 + HT. In summary, no optimal bone marrow dose/volume constraints standard has been proposed until now.

In several studies, the external contour of all the pelvis bones was delineated as TPB. The TPB was then divided into three subsites: lumbosacral spine, lower pelvis, and ilium, and the importance of these particular regions was stated [8, 21, 25]. However, in our study, all external contours of the bones within the PTV coverage were delineated as a proxy for the TPB to ensure repeatability and to avoid multiple collinearities in the multivariate analysis. Our results are consistent with previous studies, which found that patients with bigger volumes of TPB who received low-dose radiation had higher rates of HT (grade ≥ 3) [20, 28]. Patients who received TPB_V20 ≥ 71% were more likely to develop grade ≥ 3 HT (84.1% vs. 38.5%, *P* < 0.001), with specificity and sensitivity threshold of 78.1% and 71.2%, respectively. The association with the low-dose dosimetric parameter (TPB_V20) was consistent with the known radiosensitivity of bone marrow.

This study has several limitations. First, this was a single-center retrospective study with a small number of patients, which may have resulted in selection bias. Furthermore, the dosage and duration of G-CSF treatment differed with the conditions of patients, which may have affected on the changing trend of AMC, ANC, and WBC. Finally, we contoured the external contours of the bones as opposed to the actual proliferating active bone. Despite the aforementioned limitations, the present study highlights the prognostic efficacy of dosimetric parameters and AMC for HT in cervical cancer patients undergoing CCRT.

## Conclusion

The changing trend of AMC can be used as an effective predictor for the timing and severity of the ANC/WBC nadirs and prophylactic G-CSF administration. Furthermore, according to the multivariate logistic regression analysis, the chemotherapy regimen and TPB_V20 were independent risk factors for the development of grade ≥ 3 HT. Maintaining TPB_V20 < 71% and selecting single-agent cisplatin or carboplatin could significantly reduce grade ≥ 3 HT in cervical cancer patients undergoing CCRT. However, the predictive value of dosimetric parameters and AMC for acute HT requires further investigations.

## Data Availability

The datasets used during the current study are available from the corresponding author on reasonable request.

## Abbreviations

VMAT: Volumetric-modulated arc therapy
CCRT: Concurrent chemoradiotherapy
HT: Hematological toxicity
AMC: Absolute monocyte count
ANC: Absolute neutrophil count
WBC: White blood cells
IMRT: Intensity-modulated radiation therapy
TOMO: Helical tomography radiotherapy
3D-CRT: Conventional three-dimensional conformal radiotherapy
OARs: Organs at risk
TPB: Total pelvic bones
Q1: 25Th percentile
Q3: 75Th percentile
SD: Standard deviation
G-CSF: Granulocyte colony-stimulating factor
